# On Small and Repeated Bleedings in Hæmoptysis and Incipient Phthisis

**Published:** 1831

**Authors:** 


### ON SMALL AND REPEATED BLEEDINGS IN HÆMOPTYSIS AND INCIPIENT PHTHISIS. By Dr. J. CHEYNE.

[Dublin Hospital Reports, Vol. V.]

THESE observations are in the form of a letter from Dr. Cheyne to Dr. Graves, and the author commences by informing us that he has often seen phthisis usher itself in without any unequivocal symptom of pulmonary affection, but apparently as "a fever of an inflammatory kind, with quick pulse, hot skin, flushed countenance, white tongue, high-coloured urine, &c." The disease might have passed for a general fever, there being no local disease prominent. Some alleviation of symptoms usually takes place, after a period of two or three weeks, and the physician promises recovery, but is deceived, as phthisis either rapidly advances, or takes a slow but fatal course.

"In the course of such attendances, the physician at last begins to feel some surprise at the continued quickness of the pulse; he fears that all cannot be right while the patient, although he eats well and walks about, does not gain strength; the breathing too is not quite natural, an occasional dry cough occurs, of which the patient seems unconscious, and emaciation is palpable. The disease has now made some progress, and another physician being called in, the case is looked at with a new eye; night perspirations are discovered; on minute inquiry hectic fever is more than suspected, and the case is pronounced to be incipient phthisis. It is in the more chronic cases to which I have alluded, that small bleedings of six ounces practised once in four or five days have sometimes apparently proved sanative."

There is a species of hæmoptysis, says Dr. Cheyne, or rather of bronchial hæmorrhage, which runs a course of two or three weeks, and which is also attended with symptoms of general fever; but in his judgment both the fever and hæmorrhage are symptomatic of incipient consumption. In these cases recovery seems to take place under antiphlogistic treatment; but the recovery is often not solid. Gradual emaciation is observable, with that ominous dry barking cough, which is often so long a solitary symptom of slowly advancing tuberculation. After some months of declining health, the disease advances more rapidly, and hectic fever concludes the sad history. "Patients of this description may sometimes be saved by timely bleedings, not exceeding six ounces every sixth or seventh day, with a regimen suited to the strumous diathesis."

"Hæmoptysis in its more common form of pulmonary apoplexy, as it is fancifully called by the French pathologists, may sometimes be successfully treated in the same way, namely, by small bleedings repeated at stated intervals. But these are topics which would require to be more carefully handled than my time at present will permit: they would best be explained, not by the hospital physician, but by him who practises extensively among the upper classes; but unhappily they who, advancing in life, obtain lucrative employment, are often obliged to forsake the favourite pursuits of their youth for occupations neither so interesting nor agreeable, and their matured experience, which often contradicts the conclusions of youthful zeal, is unproductive to all but themselves."

Dr. Cheyne next proceeds to state a case which first confirmed his opinion respecting the applicability of small bleedings to hæmoptysis. It was that of a gentleman who had long laboured under bronchial hæmorrhage, so obstinate that it resisted all the ordinary methods of treatment. Dr. C. observed that the hæmorrhage returned upon excitement of any kind taking place. Even the flurry produced by the Doctor's carriage coming up to the door invariably caused a slight hæmoptoic attack. This suggested the employment of small artificial bleedings. The case is detailed by the patient himself, who is a clergyman. These details we shall abridge.

When about the age of 15 (in the year 1807) the hæmoptysis commenced, and continued annually from that time till 1823, in which year the attack was so serious that change of air was recommended, and he went to Nice, and ultimately on to Rome. At Rome, and afterwards at Geneva, the bleeding continued, though he had the skilful attendance of Dr. Clark. In November, 1824, he returned to Ireland, and in the course of the Winter, the complaint increased in frequency and extent of hæmorrhage. Emaciation was the consequence. In February, 1825, the plan of small bleedings was commenced. For some months previously he had daily at least three discharges of blood from the lungs.

"About the middle of February, immediately after an attack, six ounces of blood were taken from the arm. For three days after he had no attack, and on the fourth a slight one, after which six ounces of blood were again taken. No attack for ten days. The attacks now gradually became less and less frequent, but every week six ounces of blood were taken from the arm. In the beginning of May he went to the country, with directions to continue the stated bleedings, which he did regularly every week, using the lancet himself, and thus being enabled at once to check an attack. The blood was invariably much cupped and buffed; the complaint gradually subsided; health and strength slowly returned. During the whole of the ensuing Winter he was able to take exercise in the open air without suffering from cold. In the month of June, 1826, he again entered upon the duties of his profession, from which he has never since been obliged to absent himself, and with the exception of an occasional attack, which occurs generally in the Spring and Autumn, and is invariably checked by the lancet; he is now in as good health as he has ever been at any period of his life."

It is curious that till these repeated small bleedings were employed, the digestive organs were constantly deranged; but with the abatement of the hæmoptysis, the stomach began to recover its tone, and the bowels to act without opening medicine.

Of late, Dr. Cheyne has frequently prescribed venesection in the more common form of hæmoptysis, and, in some cases, with success. Unfortunately he has no notes of these cases; but some of them are distinctly in his recollection.

"In bronchial hemorrhage it is not the loss of blood which is destructive to life, but the inflammation and disorganizing process, which is caused by tubercles, of which the hemorrhage is but a symptom, and often even a means of temporary relief. And considering that not merely has the hemorrhage been checked by venesection, but the vascular irritation on which it depends, in some sort arrested, I have been led also to try small bleedings once every week or ten days, in what I conceived to be incipient phthisis, and with a degree of success which forbids the relinquishment of that practice. Among other encouraging cases, I may mention that of a young gentleman of a family which consumption had completely ravaged; he came to me last Spring with a dry barking cough, (not from cold.) There was a portion of the thorax in which respiration was inaudible, and which, on percussion, emitted scarcely any sound, and was also the seat of uneasiness; and emaciation had already commenced. This patient was relieved by these bleedings, and when I last saw him he said he was quite well, and his appearance did not contradict the assertion.

Both in hæmoptysis and in incipient phthisis, these small bleedings may be practised with safety, and often, if I mistake not, with more advantage than any other remedy in use. To acquire a just view of such cases we ought to consider them as instances of scrofula affecting the lungs, in which an inflammatory state is caused by the presence in that organ of irritating substances, as tubercles doubtless are. In phthisis, these attacks of inflammation in the tuberculated portions of the lungs precipitate disorganization. Phthisis is often, for a long time, only suspected, until uneasiness in the chest, perhaps increased frequency of the pulse, hurry of respiration, and greater debility, prove that inflammation around some clusters of tubercles is more speedily accomplishing the destiny of the patient. If the inflammation were subdued and the general health improved, perhaps it might be within the power of the absorbents to remove tubercles if still in an early stage. This view would justify the exhibition of remedies of opposite kinds. No point is better established than that the scrofulous patient is best treated by nourishing and restorative food and medicine, but there are many cases of scrofula in which we must, for a time, substitute bleeding and an antiphlogistic regimen for generous food and stimulating applications, to prevent the disorganization of a viscus, and of such cases this appears to be one.

In hæmoptysis, venesections act rather as an alterative than a styptic, mere hemorrhage from the lungs does not justify the measure. Bleeding, however, is amply justified by the existence, during hæmoptysis, of pain, hurried respiration, or any other symptom of parenchymatous or of membranous inflammation.

In cases of hæmoptysis with inflammatory symptoms, venesection may be necessary during the attack, but generally tartar emetic in nauseating doses, given every hour, or every two hours, proves a more powerful styptic: one-third or one-fourth of a grain of tartar-emetic in a draught containing also ten or fifteen grains of nitre, a combination which is often powerfully diuretic, will be still more efficacious. But if respiration be natural, and there be no cough, stricture, or pain in the thorax, the case will be better treated by small doses of opium, two or three grains of Dover's powder, for instance, every two or three hours; to minute doses of opium may be added, a couple of grains of superacetate of lead or a dose of Ruspini's styptic.

Finally, small bleedings, practised in incipient phthisis, *enable the physician more safely to enlarge the patient's diet, and to prescribe tonics, such as Griffith's mixture or Heberden's ink.* The treatment which I would recommend in incipient phthisis may be stated in a few lines. Journeying, if practicable, or what is better still, in fine weather going from shore to shore in the steamers, short residences at Mallow, or the Cove of Cork, or some favourite spot in England, or, during the Summer, in Scotland. Diet as generous as the state of the lungs will permit, in some cases a glass or two of claret, and small bleedings. Sponging the chest and arms with very dilute nitro-muriatic acid, or with five parts of Mindererus's spirit, and one of spirit of rosemary: an issue over the most suspected portion of the lungs, or a succession of blisters, after each bleeding, not much larger than a dollar. A light bitter two or three times a day, with twenty or thirty drops of laurel water, or the nitro-muriatic acid internally, or perhaps some preparation of iron. If I had time I would explain my reasons for rarely sending patients, in any stage of consumption, to the continent of Europe."

Whatever may be Dr. Cheyne's reasons for this resolution, there is reason in it. We too are of the same opinion, and our reasons will be before the public by the time this Number sees the light.*

* See Dr. Johnson on "CHANGE of AIR," Part the Third, on the "Moral, Physical, and Medicinal Effects of an Italian Climate."

